# Managing Zinc Supplementation in Hemodialysis Patients: Balancing and Preventing Deficiencies in Serum Copper and Zinc Levels with and Without HIF-PH Inhibitors

**DOI:** 10.3390/nu16234135

**Published:** 2024-11-29

**Authors:** Akira Takahashi

**Affiliations:** Dialysis Center, Tesseikai Neurosurgical Hospital, 28-1 Nakanohonmachi, Shijonawate 575-8511, Japan; kinnereth@gaia.eonet.ne.jp; Tel.: +81-72-877-6639; Fax: +81-72-877-6692

**Keywords:** copper deficiencies, hemodialysis, hypoxia-inducible factor-prolyl hydroxylase inhibitors, zinc deficiency, zinc supplementation

## Abstract

Background/Objectives: Zinc supplementation induces metallothionein, leading to reduced serum copper levels. Conversely, serum copper concentrations tend to rise with the use of HIF-PH inhibitors. Methods: To establish a safe level of zinc supplementation that avoids copper deficiency, serum copper and zinc concentrations measured every three months were retrospectively analyzed over five years in 50 patients undergoing hemodialysis. Results: At the initiation of the study, the median (IQR) concentrations were 100 (84.25–109) µg/dL for serum copper and 60.5 (50.5–70) µg/dL for serum zinc. All participants without zinc supplementation exhibited zinc deficiency (<80 µg/dL). After three months, copper deficiency (<71 µg/dL) was observed when serum copper concentrations were <98.6 µg/dL for patients with HIF-PH inhibitors and <90.3 µg/dL for patients without them. Reduced zinc supplementation may be necessary when serum copper falls below 90 µg/dL. Zinc levels remained deficient because supplementation was limited due to concerns about copper deficiency. Lowering the target zinc level to around 80 µg/dL instead of the conventional 80–120 µg/dL may be safer. Conclusions: Regular monitoring of both copper and zinc levels, taking place at least every three months, is recommended to adjust zinc supplementation, especially in patients on HIF-PH inhibitors. Copper supplementation should also be considered alongside zinc supplementation to effectively treat hypozincemia.

## 1. Introduction

In patients undergoing hemodialysis, serum zinc levels often decrease due to inadequate zinc intake, impaired absorption, and excessive zinc loss, with most patients exhibiting hypozincemia [1]. To address this, our hospital has provided zinc supplementation therapy since 2018. However, zinc supplementation can lower serum copper levels by inducing metallothionein [2], necessitating the establishment of safe supplementation methods that prevent hypocupremia.

Looking toward future measures in dialysis treatment, the latest statistical data from the 2022 Annual Dialysis Data Report by the Japanese Society for Dialysis Therapy (JSDT) highlight the need to address infectious diseases, cardiovascular disease, and aging-related issues [3]. However, the interpretation of these data is the responsibility of the author and does not reflect the official policy or interpretation of the JSDT. Zinc plays a crucial role in combating these challenges: strengthening the immune system and improving renal anemia to fight against infectious disease, preventing vascular calcification to address cardiovascular risks, and mitigating sarcopenia and dementia to combat aging. While numerous factors contribute to these conditions, zinc is particularly important.

As a measure against infectious diseases, zinc exhibits anti-inflammatory and antioxidant effects and is essential for the function of enzymes such as superoxide dismutase and glutathione peroxidase [4]. Some studies have shown that zinc is critical for the transcriptional activity of Nrf2 [5], and a meta-analysis reported that zinc supplementation may improve serum CRP levels in patients with renal failure [6]. During hematopoiesis, zinc is required in processes involving GH, IGF-1, and GATA-1, and adequate supplementation can reduce the required dosage of ESA [7]. However, when using HIF-PH inhibitors, zinc inhibits HIF, so zinc supplementation does not affect dosage requirements [8].

For cardiovascular disease, zinc has been shown to suppress phosphate-induced vascular calcification [9]. Furthermore, because zinc inhibits HIF-1α-induced calcification of vascular smooth muscle cells, maintaining appropriate serum zinc concentrations is particularly important when treating renal anemia with HIF-PH inhibitors [10].

Regarding neurodegenerative conditions, researchers have raised concerns about copper-induced neurodegeneration contributing to Alzheimer’s disease [11]. Monitoring and stabilizing serum copper and zinc concentrations may help prevent Alzheimer’s disease [12]. To avoid excessive increases in serum copper caused by HIF-PH inhibitors, combining them with zinc supplementation, which lowers serum copper levels, is crucial [13].

Given this background, I believe that maintaining normal serum zinc and copper concentrations could play an important role in improving the prognosis of hemodialysis patients. By identifying the conditions under which zinc supplementation induces hypocupremia, we can establish safe supplementation methods and strategies for addressing potential deviations from the normal range.

Nishime et al. state that safe, non-hypocupremia-causing serum zinc concentrations in dialysis patients are in the range of 41.3–78.3 µg/dL, while serum copper concentrations are in the range of 66.5–96.5 µg/dL [14]. Because 60–80% of blood zinc is bound to albumin [15], it may be difficult to achieve normal serum zinc concentrations of 80 µg/dL or higher in dialysis patients, who tend to have low serum albumin levels.

The purpose of this study was to retrospectively investigate serum zinc and copper concentrations in hemodialysis patients after zinc supplementation therapy and clarify the conditions necessary to maintain zinc and copper concentrations within the normal range. Furthermore, strategies for addressing deviations from the normal range were discussed, along with exploring new treatments for cases that cannot be managed with existing methods.

## 2. Methods

This is a retrospective cohort study of routine (or real-world) clinical practice.

### 2.1. Participants

At our hospital, only serum zinc levels were initially measured for patients who required zinc supplementation. Nonetheless, due to the observation of hypocupremia eight months after the start of zinc supplementation, simultaneous measurement of serum copper and zinc concentrations began in August 2018. Simultaneous measurements were initiated in 24 patients undergoing morning hemodialysis, and one year later, the number of patients undergoing morning measurements had increased to 54. Since four patients dropped out due to being transferred to another hospital or death, the study included 50 outpatients undergoing morning hemodialysis enrolled over a five-year period from August 2019 to May 2024. It has been reported that serum zinc concentrations are typically lower when measured in the afternoon [16]. Serum zinc concentrations were, on average, 5 µg/dL (7%) lower in afternoon dialysis patients compared to morning patients, so only morning hemodialysis patients were included.

The basic information of participants during the observation period up to August 2024 is shown in Table 1.

Participants received only general dietary advice for dialysis diets, with no specific recommendations provided regarding zinc and copper intake. This study was approved by the ethics committee of the hospital, and written informed consent was obtained from all participants.

### 2.2. Measurements

The data collected from participants included measurements of serum zinc and serum copper concentrations. Also recorded were whether participants were using HIF-PH inhibitors at the time of blood collection and the dosage. Additional information gathered included the examination date, sex, age, history of dialysis, underlying disease that led to dialysis initiation, name of the zinc supplement used, dosage, date of zinc intake, and any comorbid conditions (such as surgeries or infections) for each patient. In total, 968 test points were obtained from patients who underwent simultaneous measurement of serum copper and zinc concentrations between August 2018 and May 2024. These data excluded test values recoded during hospitalization due to COVID-19, issues with vascular access, fractures, and other complications.

### 2.3. Measurements of Serum Zinc and Copper Concentrations

The serum zinc and copper concentrations of the participants were measured every three months as part of routine clinical practice. Serum zinc concentrations were measured using a direct colorimetric assay, following the nitro-PAPS method, and using JCA-BM6050 BioMajesty (JEOL Ltd., Tokyo, Japan) and ESPA ZnII (Nipro Co., Ltd., Osaka, Japan) reagents. The serum copper concentration was measured using a direct colorimetric assay, following the 3,5-DiBr-PAESA method, and using JCA-BM6050 BioMajesty and Quick Auto Neo Cu (Shino-Test, Tokyo, Japan) reagents. Normal ranges for the serum copper and zinc concentrations were 71–132 µg/dL and 80–130 µg/dL, respectively [17,18].

### 2.4. Statistical Analysis

Data for continuous variables were presented as the mean ± standard deviation for normally distributed data and the median (interquartile range) for non-normally distributed data. Data for categorical variables were presented as percentages with counts. Microsoft 365 Excel (Microsoft Corporation, Redmond, WA, USA) was used for data analysis. The baseline characteristics of the two groups were compared using histograms to determine whether they were normally distributed, and we then used a Mann–Whitney U test for non-normally distributed data. Categorical data were compared using a chi-square test. Statistical significance was set at *p* < 0.05.

### 2.5. Treatment of Zinc Deficiency

The following method was adopted as an appropriate zinc supplementation method to prevent copper deficiency in hemodialysis patients [7]. Before zinc supplementation, serum zinc and copper concentrations were measured simultaneously, and if copper deficiency was found, treating it was prioritized. Even after starting zinc supplementation, the amount of zinc supplementation was adjusted, and serum copper and zinc concentrations were measured simultaneously at least every three months. Zinc supplementation was initiated using 50 mg/day of zinc acetate hydrate (molecular formula: C_4_H_6_O_4_Zn.2H_2_O, zinc content 50 mg) for the indication of hypozincemia. For participants with gastrointestinal symptoms, 150 mg/day of polaprezinc (molecular formula: C_9_H_12_N_4_O_3_Zn, zinc content 34 mg) was used as a zinc-containing stomach medicine, even though there was no indication for hypozincemia.

The patients were treated with darbepoetin alfa (Kyowa Kirin Co., Ltd., Tokyo, Japan; injection at doses of 10, 20, and 30 µg) or roxadustat (Astellas Pharma Inc., Tokyo, Japan; tablets at doses of 20, 50, and 100 mg) as an HIF-PH inhibitor. The only information collected outlined whether or not HIF-PH inhibitors were used and, if so, the dates and times at which they were used.

The initial zinc supplementation protocol used in this study set a target serum zinc concentration of 80 to 120 µg/dL. If the concentration reached 100 µg/dL or higher, zinc supplementation was reduced. If it reached 120 µg/dL or higher, a break in medication was performed.

After simultaneous measurement of serum copper and zinc concentrations was initiated, some patients with serum copper concentrations below 90 µg/dL had hypocupremia when tested three months later. Therefore, an additional rule was created: if the serum copper concentration fell below 90 µg/dL, zinc supplementation was reduced.

## 3. Results

### 3.1. Baseline Characteristics of Participants with and Without Hypocupremia

In total, 20 out of 50 participants experienced periods of hypocupremia. Regarding baseline characteristics, participants who developed hypocupremia tended to be older, having a median age of 79.9 years old. There were no significant differences in the underlying disease that necessitated dialysis between participants who developed hypocupremia and those who did not develop it. The basic information of patients who developed hypocupremia and those who did not do so is shown in Table 2.

Hypocupremia was observed in one patient with each of the following comorbid conditions: pulmonary edema, post-gastrectomy, and chemotherapy. Nine cases were also observed in patients using HIF-PH inhibitors, of whom two participants developed hypocupremia because they had not reduced their zinc dosage despite discontinuing the HIF-PH inhibitor. These are shown in Table 3.

### 3.2. Serum Copper and Zinc Concentrations of Each Participant at the Start of Simultaneous Measurement

When simultaneous measurement of serum copper and zinc concentrations was started for each participant, the median (IQR) was 100 (84.25–109) µg/dL of serum copper and 60.5 (50.5–70) µg/dL of serum zinc. All participants who did not receive zinc supplementation had hypozincemia (<80 µg/dL).

**Figure 1:**
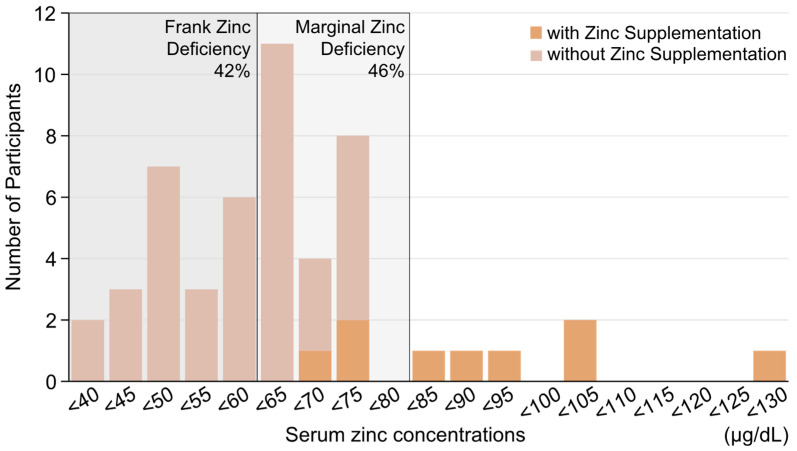
Histogram of serum zinc concentrations when simultaneous measurement of the serum zinc and copper concentrations of each participant was initiated. Frank Zinc Deficiency is less than 60 µg/dL, while Marginal Zinc Deficiency is 60–80 µg/dL. Serum zinc concentration (normal range 80–130 µg/dL).

Figure 1 shows a histogram of serum zinc concentrations that details when simultaneous measurement of the serum zinc and copper concentrations of each participant was initiated.

### 3.3. Reasons for Changing the Zinc Supplement Dose

Of the 968 times that serum copper and zinc concentrations were measured simultaneously, when zinc supplementation was reduced or discontinued, the serum copper concentration was 74.2 ± 26.4 µg/dL and the serum zinc concentration was 92.6 ± 30.0 µg/dL. When zinc supplementation was increased, the serum copper concentration was 104.6 ± 29.7 µg/dL and the serum zinc concentration was 58.2 ± 11.9 µg/dL. When the amount of zinc supplementation was not changed, the serum copper concentration was 99.5 ± 20.2 µg/dL and the serum zinc concentration was 75.8 ± 17.8 µg/dL. The reasons for changes in zinc supplementation and the percentages of those changes are shown in Table 4.

In the zinc supplementation protocol mentioned above, the only factor considered for reducing zinc supplementation was the serum zinc level. However, this retrospective analysis revealed that a reduction in zinc supplementation was 3.7 times more likely to occur due to a decrease in serum copper concentration than an increase in serum zinc concentration. Furthermore, despite the presence of hypozincemia, 16.1% of patients did not increase their zinc dosage due to hypocupremia or decreasing serum copper levels. This finding highlights the need to investigate the status of serum copper concentration three months prior to the onset of hypocupremia.

### 3.4. Serum Copper and Zinc Concentrations Three Months Prior to the Onset of Hypocupremia

When HIF-PH inhibitors were used, the serum copper concentration three months prior to the onset of hypocupremia (<71 µg/dL) was 98.6 ± 21.6 µg/dL or less, while the serum zinc level was 76.9 ± 21.0 µg/dL or more. When HIF-PH inhibitors were not used, the serum copper concentration three months prior to the onset of hypocupremia was 90.3 ± 16.8 µg/dL or less, and the serum zinc level was 71.4 ± 20.4 µg/dL or more. Therefore, if serum copper concentrations fell below these levels or serum zinc concentrations rose above these levels, it was possible that serum copper concentrations would fall below the lower limit of the normal range of 71 µg/dL after three months.

### 3.5. The Achievement Rates of Normal Serum Zinc and Copper Concentrations

Of the 968 test results obtained from 50 participants, the achievement rate for normal serum zinc and copper concentrations was 27.5%.

### 3.6. All Tests Were Divided into Four Zones According to the Lower Limits for Normal Serum Zinc and Serum Copper Concentrations

**Figure 2:**
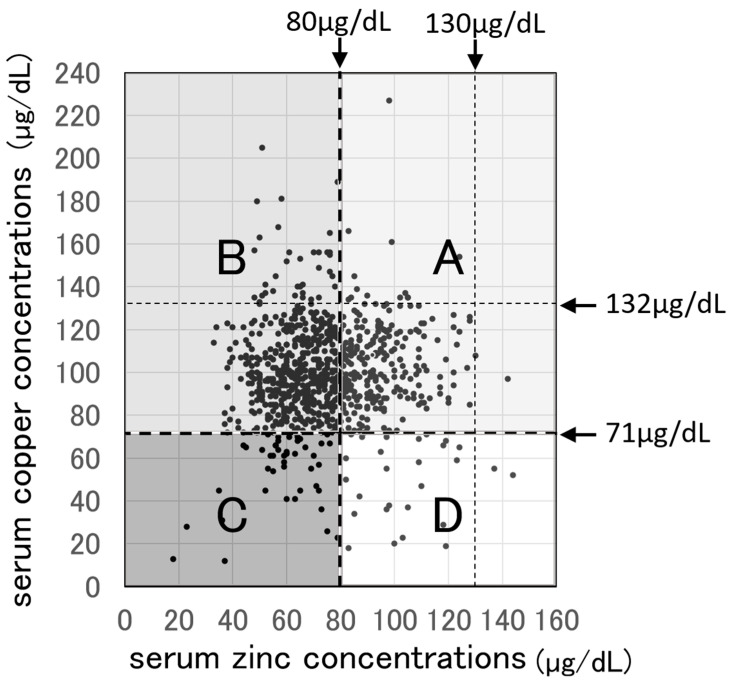
Scatterplots of 968 serum copper and zinc concentrations measured simultaneously in 50 participants over five years, divided into four zones according to the lower limit of normal serum zinc and serum copper concentrations. Zone A is the area free of hypocupremia and hypozincemia, accounting for 31.9% of the total data. Zone B is the area with hypercupremia and hypozincemia, accounting for 58.5% of the total data, and it is the largest area. Zone C is the area of hypozincemia or hypocupremia, accounting for 5.7% of the total data. Zone D is the area of hypocupremia and free of hypozincemia, accounting for 3.9% of the total data. [Cu], serum copper concentration (normal range 71–132 µg/dL); [Zn], serum zinc concentration (normal range 80–130 µg/dL).

Figure 2 shows a scatterplot of 968 simultaneously determined serum copper and zinc concentrations sourced from 50 participants over five years. This graph is divided into four zones based on the lower limit lines for serum copper levels (71 µg/dL) and serum zinc levels (80 µg/dL). Zone B contains 4.2% of participants who had hypozincemia but could not start or increase zinc supplementation due to nausea or loss of appetite. Furthermore, this zone includes 48.9% of cases in which active zinc supplementation was not administered due to concerns about hypocupremia. In Zone C, there are cases in which zinc supplementation could not be increased or initiated due to pre-existing hypocupremia. Zone D also includes cases of hyperzincemia where zinc supplementation had to be reduced.

## 4. Discussion

After simultaneous measurement of serum copper and zinc concentrations was first performed, the target serum zinc concentration for zinc supplementation in patients undergoing hemodialysis was set at 80–120 µg/dL for five years to prevent hypocupremia. If serum zinc levels reached 100 µg/dL or higher, zinc supplementation was reduced, and if serum zinc levels reached 120 µg/dL or higher, supplementation was suspended. However, only 31.9% of all test results showed no hypozincemia or hypocupremia, and the achievement rates for both normocupremia and normozincemia were low at 27.5%.

This result is related to earlier observations made when we started simultaneously measuring serum copper and zinc concentrations. Some patients who had serum copper concentrations below 90 µg/dL were found to have hypocupremia when measured three months later. A total of 24.6% of cases involved reducing or hesitating to increase zinc supplementation despite hypozincemia. Furthermore, in 24.0% of cases, the amount of zinc supplementation was not increased despite the presence of hypozincemia due to nausea and anorexia. In fact, in this study, the average serum copper concentration three months before hypocupremia onset when not using an HIF-PH inhibitor was 90.3 µg/dL. If zinc supplementation is continued in the future, it will be necessary to consider reducing or stopping zinc supplementation if the serum copper concentration is below 90 µg/dL. If zinc supplementation is continued despite the possibility of causing hypocupremia, it is necessary to simultaneously measure serum copper and zinc concentrations monthly rather than every three months.

In addition, patients who used HIF-PH inhibitors to treat renal anemia developed hypercupremia due to copper absorption promotion by HIF-PH inhibitors [13]. For this reason, zinc supplementation was set at a high level, but there were three cases in which hypocupremia occurred because zinc supplementation was not reduced after the HIF-PH inhibitor was discontinued. When using HIF-PH inhibitors, sufficient zinc supplementation was necessary to prevent vascular calcification [10] and hypercupremia [8], but it was confirmed that if HIF-PH inhibitor supplementation was reduced or discontinued for some reason, it was necessary to also reduce or discontinue zinc supplementation.

### 4.1. Treatment for Hypocupremia

As a treatment for hypocupremia, oral administration of 1.5 to 3 mg of copper per day (usually as copper sulfate) has been described [19], but as there are no pharmaceutical drugs available, non-pharmaceutical supplements are the treatment of choice. In addition, if available, injections of copper histidine [20,21] can also be used. In addition, since most dialysis patients who develop copper deficiency are also malnourished [22], hypocupremia can be reversed in about one month by adding a trace element preparation for a high-calorie infusion as part of IDPN (intradialytic parenteral nutrition) [23]. For long-term use, a manganese-free trace element preparation should be used [24]. Copper-containing nutritional supplements are sometimes used for patients with copper deficiency, but because they contain high phosphorus concentrations, dialysis patients must be careful when using them because they may develop hyperphosphatemia. The recommended dietary intake of copper is 0.9 mg (ages 18–29 and 50 and over) and 1.0 mg (ages 30–49) for adult men, and 0.8 mg (ages 18–69) and 0.7 mg (ages 70 and over) for adult women. Foods high in copper include liver (5.30 mg per 100 g of beef liver), cocoa (0.55 mg per 100 g of unsweetened baking chocolate), potatoes (0.08 mg per 100 g), mushrooms (0.50 mg per 100 g of dried shiitake mushrooms), nuts (1.89 mg per 100 g of cashews), and yuba (tofu skin, 0.60 mg per 100 g). These foods are high in copper, as well as phosphorus and potassium, so hemodialysis patients should consume them with caution [25,26,27]. Additionally, squid, octopus, shrimp, crabs, and shellfish transport oxygen through copper-containing hemocyanin [28]. These creatures not only contain a lot of copper but also have high phosphorus contents, so similar caution is required.

### 4.2. Strategies for Maintaining Normal Zinc and Copper Serum Levels in the Future

In this analysis, the mean serum zinc level three months before the onset of hypocupremia (defined as serum copper levels <71 µg/dL) was ≥76.9 µg/dL with HIF-PH inhibitors and ≥71.4 µg/dL without them. These values are below the normal lower limit for zinc concentrations of 80 µg/dL. If zinc supplementation continues targeting serum zinc levels of 80–120 µg/dL, as per the current protocol, there is a high risk of developing hypocupremia. To mitigate this, the target zinc concentration should be adjusted to around 80 µg/dL, rather than the conventional 80–120 µg/dL, as the upper target value may be unnecessarily high.

When not using HIF-PH inhibitors, zinc supplementation should be reduced if serum zinc concentrations reach 70 µg/dL or higher. When using HIF-PH inhibitors, reduction should occur at 75 µg/dL or higher. Zinc supplementation should be discontinued entirely if serum zinc levels reach 80 µg/dL, which represents the lower limit for normal zinc concentrations.

As mentioned in the Introduction, a normal serum zinc concentration of 80 μg/dL may need to be re-evaluated for hemodialysis patients, given their high prevalence of hypoalbuminemia. Equations for calculating albumin-corrected zinc concentrations, similar to those for calcium correction, have been reported [29,30]. However, factors such as dietary phytic acid may still lead to hypozincemia even when albumin levels are normal. Thus, further validation is necessary before adopting this method widely. For now, a target serum zinc concentration of less than 80 µg/dL appears appropriate. Additionally, if serum copper levels fall below 90 µg/dL, zinc supplementation should be discontinued.

In Zone C of Figure 2, 24.6% of patients exhibited hypozincemia but refrained from increasing zinc supplementation due to concerns about hypocupremia. For patients eligible to use HIF-PH inhibitors for renal anemia treatment, combining zinc supplementation with HIF-PH inhibitors offer a way to manage both hypozincemia and hypocupremia [13]. Furthermore, combining zinc supplementation with copper supplements could allow more efficient and active zinc supplementation. 

In Zone B, 55.2% of patients (24% of the total) experienced hypozincemia but could not start or increase zinc supplementation due to nausea or loss of appetite. Recently available oral zinc histidine supplements, which cause fewer gastrointestinal symptoms due to the stable bond between zinc and histidine, offer a promising alternative. Switching from conventional oral medications, such as zinc acetate, to zinc histidine could alleviate gastrointestinal side effects, enabling adequate zinc intake.

This study was a retrospective clinical observation conducted under a protocol that initially only considered serum zinc levels, without addressing serum copper concentrations. After identifying cases where serum copper levels fell below 90 µg/dL at baseline and where hypocupremia developed within three months, serum copper levels were added as a protocol condition.

However, follow-up observations were not conducted under a consistent treatment plan. Prospective trials incorporating this updated protocol are needed in the future. It is hoped that safe and effective zinc supplementation, which improve the prognosis of dialysis patients, can be widely implemented.

This study focused solely on individual zinc and copper concentrations. Previous research has shown that reducing the copper-to-zinc ratio through zinc supplementation can lower the CRP-to-albumin ratio, improving nutritional, oxidative, and inflammatory statuses [31]. This warrants further investigation. The findings from this study are expected to inform future interventional studies to determine optimal zinc dosage protocols. However, this study did not examine whether maintaining both zinc and copper concentrations within the normal range enhances these outcomes. The findings from this study are expected to inform future interventional studies to determine optimal zinc dosage protocols.

## 5. Conclusions

To prevent hypocupremia in dialysis patients receiving zinc supplementation, it is essential to measure serum copper and zinc concentrations simultaneously at least every three months, focusing not only on serum zinc but also on serum copper concentrations. Additionally, since HIF-PH inhibitors can increase serum copper concentrations, zinc supplementation should be carefully adjusted in consideration of their use.

The following guidelines have been derived as strategies to prevent hypocupremia in hemodialysis patients: Zinc supplementation should be reduced when serum zinc levels reach 70 µg/dL or higher without HIF-PH inhibitors, and 75 µg/dL or higher with HIF-PH inhibitors. Zinc supplementation should be discontinued entirely if serum zinc levels reach 80 µg/dL, the lower limit of the normal range for zinc. Caution should be exercised when administering zinc supplementation to patients with serum copper concentrations below 90 µg/dL.

To ensure effective and safe zinc supplementation, future considerations should include a combination of zinc and appropriate copper supplementation.

## Figures and Tables

**Table 1:** The baseline characteristics of participants.

Number of Participants	50
Female sex, *n* (%)	23 (46.0)
Age, median (IQR) (years)	79.9 (75.1–83.0)
Duration of Hemodialysis, median (IQR) (years)	6.5 (3.9–10.2)
Etiology, n (%)	
Nephrosclerosis	19 (38.0)
Diabetic nephropathy	20 (40.0)
Chronic glomerulonephritis	5 (10.0)
Others	6 (12.0)

Data for continuous variables are presented as median (interquartile range) values for non-normally distributed data. Data for categorical variables are presented as percentages with counts. IQR, interquartile range.

**Table 2:** Baseline characteristics of participants with and without hypocupremia.

	With Hypocupremia	Without Hypocupremia	*p*-Value
Number of participants	20 (40.0)	30 (60.0)	
Female sex, *n* (%)	10 (50.0)	13 (43.3)	0.643
Age, median (IQR) (years)	79.9 (75.1–83.0)	74.6 (65.6–82.1)	0.056
Duration of Hemodialysis, median (IQR) (years)	7.2 (4.6–13.1)	5.5 (3.4–8.2)	0.197
Etiology, *n* (%)			0.098
Diabetic nephropathy	10 (50.0)	9 (30.0)	
Nephrosclerosis	9 (45.0)	11 (36.7)	
Chronic glomerulonephritis	1 (5.0)	4 (13.3)	
Others	0 (0.0)	6 (20.0)	
Combined use of HIF-PH inhibitors, *n* (%)	9 (45.0)	0 (0.0)	<0.05

Data for continuous variables are presented as the median (interquartile range) for non-normally distributed data. Data for categorical variables are presented as percentages with counts. The baseline characteristics of the two groups were compared using a Mann–Whitney U test for non-normally distributed data. Categorical data were compared using a chi-square test. Statistical significance was set at *p* < 0.05. HIF-PH, hypoxia-inducible factor–prolyl hydroxylase; IQR, interquartile range.

**Table 3:** Comorbidities in patients with hypocupremia.

Comorbidities	*n* (%)
Zinc dosage continued after HIF-PH inhibitors were discontinued	2 (10.0)
After gastrectomy	1 (5.0)
Hospitalized for pulmonary edema	1 (5.0)
During anti-cancer chemotherapy	1 (5.0)
Nausea, decreased food intake	4 (20.0)

Data for categorical variables are presented as percentages with counts.

**Table 4:** Reasons for changes in zinc supplementation and the proportion of each change out of 968 test points.

Zinc Supplementation *n* (%)	Reasons for Changing or Not Changing Zinc Supplementation	Number of Times *n* (%)
Dose reduction or discontinuation 122 (12.6)	[Zn] > 100 μg/dL	22 (2.3)
	[Cu] < 71 μg/dL or decreasing trend	82 (8.5)
	Nausea, anorexia	19 (2.0)
Increased dosage 138 (14.3)	Hypozincemia	119 (12.3)
	Hypercupremia	11 (1.1)
	Combined use of HIF-PH inhibitor and zinc supplementation to treat hypozincemia and hypocupremia	8 (0.8)
No change in dosage 708 (73.1)	Despite the presence of hypozincemia	
	[Cu] < 71 μg/dL or decreasing trend	156 (16.1)
	Nausea or loss of appetite	213 (22.0)
	Other, unknown.	339 (35.0)

Data for categorical variables are presented as percentages with counts. [Cu], serum copper concentration (normal range 71–132 µg/dL); [Zn], serum zinc concentration (normal range 80–130 µg/dL).

## Data Availability

The data that support the findings of this study will be made available by the corresponding author upon reasonable request.

## Abbreviations

CRP	C-reactive protein
Cu	copper
[Cu]	serum copper concentrations
ESA	erythropoiesis-stimulating agent
GH	growth hormone
HIF	hypoxia-inducible factor
HIF-PH	hypoxia-inducible factor-prolyl hydroxylase
IGF-1	insulin-like growth factor-1
IQR	interquartile range
Nrf2	nuclear factor erythroid 2-related factor 2
Zn	zinc
[Zn]	serum zinc concentrations
